# LncRNA SNHG3 promotes gastric cancer cell proliferation and metastasis by regulating the miR-139-5p/MYB axis

**DOI:** 10.18632/aging.203732

**Published:** 2021-12-13

**Authors:** Yan Xie, Li Rong, Min He, Yuyou Jiang, Haiyu Li, Li Mai, Fangzhou Song

**Affiliations:** 1 Molecular Medicine and Cancer Research Center, Chongqing Medical University, Chongqing 400016, China; 2 Qinggang Senior Care Center, The First Affiliated Hospital of Chongqing Medical University, Chongqing 400016, China; 3 Department of Gastroenterology, Bishan Hospital of Chongqing, Chongqing 402760, China; 4 Chongqing Public Health Medical Center, Chongqing 400036, China; 5 Department of Laboratory Medicine, The Second Affiliated Hospital of Chongqing Medical University, Chongqing 400010, China

**Keywords:** gastric cancer, SNHG3, miR-139-5p, MYB

## Abstract

The long non-coding RNA (lncRNA) SNHG3 has been shown to play oncogenic roles in several cancer types, but the mechanisms underlying its activity are poorly understood. In this study, we aimed to explore the clinical relevance and mechanistic role of SNHG3 in gastric cancer (GC). We found that SNHG3 expression in GC cell lines and tissues was significantly increased, and the upregulation of this lncRNA was correlated with tumor clinical stage and decreased patient survival. Knocking down SNHG3 in GC cells impaired the proliferative, migratory, and invasive activity *in vitro* and constrained *in vivo* GC xenograft tumor growth. Mechanistically, SNHG3 was found to bind and sequester miR-139-5p, thereby indirectly promoting the upregulation of the miR-139-5p target gene MYB. These data demonstrated that SNHG3 functions in an oncogenic manner to drive GC proliferation, migration, and invasion by regulating the miR-139-5p/MYB axis.

## INTRODUCTION

Gastric cancer (GC) is the third most prevalent form of cancer globally [1–3]. The majority of GC patients are diagnosed with advanced disease as it causes few symptoms in its early stages [1]. While many efforts have been made to improve GC diagnosis and treatment, patients in most countries still exhibit a 5-year survival rate of under 30% [4, 5]. It is thus vital that the mechanistic basis for GC onset and progression be more fully clarified in order to identify more reliable diagnostic/prognostic biomarkers and therapeutic targets associated with this deadly disease.

Recent advances in sequencing technologies have indicated that the majority of the human genome is made up of non-coding RNAs (ncRNAs), with long ncRNAs (lncRNAs) being those > 200 nucleotides in length [6]. These lncRNAs can regulate diverse cellular processes under physiological and pathological conditions, contributing to oncogenesis and tumor malignancy [7]. MicroRNAs (miRNAs) are small (20-25 nucleotide) ncRNAs that suppress mRNA translation by binding to the 3’- UTRs of these target mRNAs [8]. Research suggests that lncRNAs can bind to these miRNAs via sequence complementarity, serving as competing endogenous RNAs (ceRNAs) that thereby disrupt the ability of given miRNA to suppress the expression of its target mRNA [9–11].

Small nucleolar RNA host genes (SNHGs) are lncRNAs that have been shown to sequester certain miRNAs via a ceRNA mechanism, thereby leading to the indirect upregulation of a range of oncogenic genes [12]. These SNHG family members can also induce epigenetic changes in cells, directly bind to mRNAs, and alter protein stability by preventing ubiquitination [13]. SNHG3 is encoded at position 1p35.3 in humans [13], and it has been shown to be upregulated in many cancer types suggesting a potential oncogenic role for this lncRNA. For example, Ma et al. found that SNHG3 was able to promote breast cancer cell proliferative and invasive activity whereas knocking down this lncRNA enhanced triple-negative breast cancer cell malignancy [14, 15]. Zheng et al. determined that SNHG3 controls osteosarcoma cell invasive and migratory activity through the miR-151a-3p/RAB22A axis [16], whereas Fei et al. observed that this lncRNA was able to silence p21 and KLF2 and to thereby drive glioma malignancy and progression [17]. Zhang et al. determined that SNHG3 was able to regulate the miR-128/CD151 axis in hepatocellular carcinoma cells so as to enhance the induction of the epithelial-mesenchymal transition while additionally promoting sorafenib resistance [18]. Sui et al. found that SNHG3 was further able to regulate the miR-214-30/PSDM10 axis to drive the progression of papillary thyroid carcinoma [19]. In addition, recent work suggests that SNHG3 overexpression can promote GC development [20, 21], suggesting that this lncRNA may play a key role in GC progression. The mechanistic basis whereby SNHG3 influences GC, however, remains to be firmly defined.

Herein, we initially leveraged the TCGA database to conduct bioinformatics analyses identifying SNHG3 as an lncRNA closely associated with GC. We then endeavored to more fully elucidate the regulatory role of SNHG3 in the context of GC onset and progression.

## RESULTS

### Gastric cancer cells and tissues exhibit SNHG3 upregulation correlates with poor prognosis

**Figure 1:**
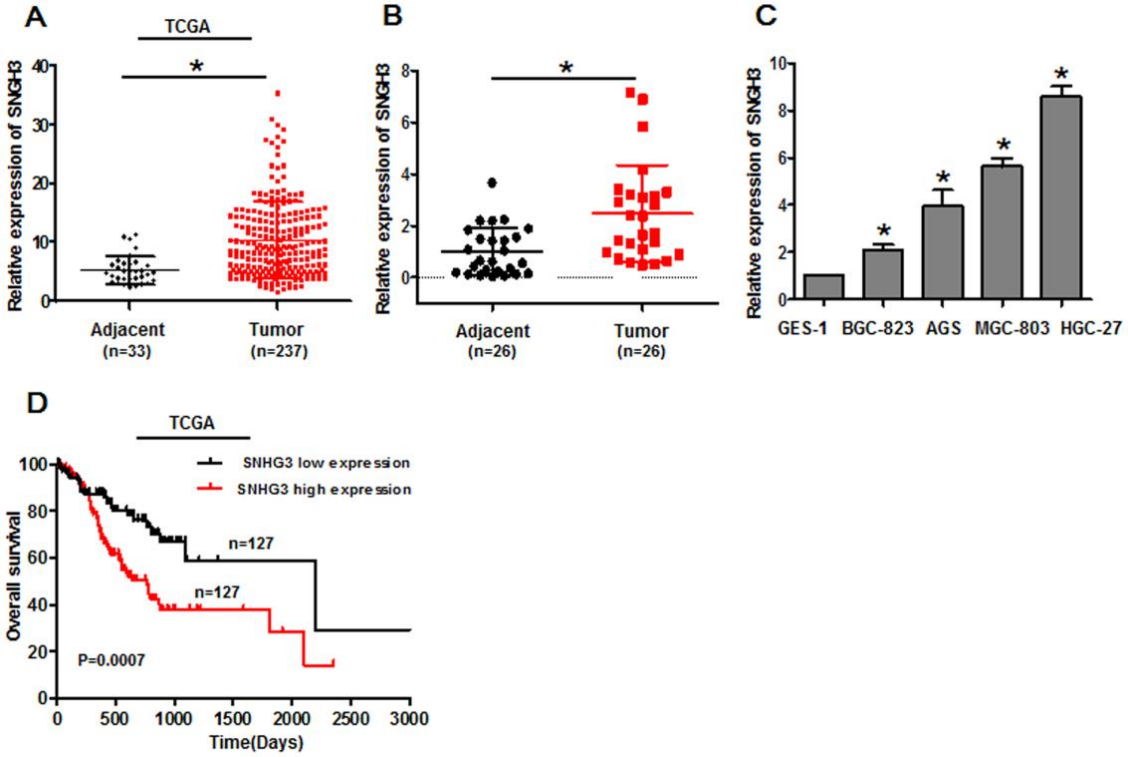
**Gastric cancer cells and tissues exhibit SNHG3 upregulation that is correlated with poor patient prognosis.** (**A**) SNHG3 expression was assessed in gastric cancer tissues in the TCGA database, *P<0.01. (**B**) SNHG3 expression was assessed in 26 paired gastric cancer tumor and paracancerous tissues via qPCR, *P<0.01. (**C**) SNHG3 expression was assessed via qPCR in gastric cancer cells (BGC-823, AGS, MGC-803, and HGC-27) and control GES-1 cells. *P<0.01 vs. GES-1 cells. (**D**) Gastric cancer patient survival for individuals in the TCGA database was assessed via the Kaplan-Meier method (log-rank, *P*=0.0007).

To explore the functional relevance of SNHG3 in the context of GC, we began by analyzing data pertaining to 237 STAD tumor tissue samples and 33 paracancerous tissue samples in the TCGA database, revealing significant increases in the expression of this lncRNA in GC tumor tissues relative to noncancerous tissues (Figure 1A). To expand upon this analysis, we then measured SNHG3 levels in 26 pairs of GC tumor and paracancerous tissues, in four GC cell lines (BGC-823, AGS, MGC-803, and HGC-27), and in control GES-1 cells via qPCR. Consistent with the TCGA data, SNHG3 expression levels were higher in tumor tissues relative to paracancerous samples (Figure 1B), and in GC cell lines relative to GES-1 controls (Figure 1C). For tumor tissues of GC patients, median SNHG3 expression levels were next used to stratify GC patients into an SNHG3-high and an SNHG3-low group. The expression of SNHG3 was closely related to tumor clinical stage (Table 1). Kaplan-Meier analyses of GC patients in the TCGA revealed that higher SNHG3 expression was related to worse survival outcomes (Figure 1D).

**Table 1:** Correlation between SNHG3 and clinical features of gastric cancer patients.

**Characteristics**	**SNHG3 expression**		***p*-Value**
	**Low**	**High**	
**Age(y)**			
<60	3	4	1.000
≥60	10	9	
**Gender**			
Male	7	10	0.411
Female	6	3	
**Clinical stage**			
I-II	8	1	0.011*
III-IV	5	12	
**Pathologic differentiation**			
No(undifferentiated)	6	9	1.000
Yes(differentiated)	7	4	
**Lymphatic invasion**			
No	7	3	0.226
Yes	6	10	

*P<0.05.

### SNHG3 knockdown suppresses gastric cancer cell growth and metastasis

**Figure 2:**
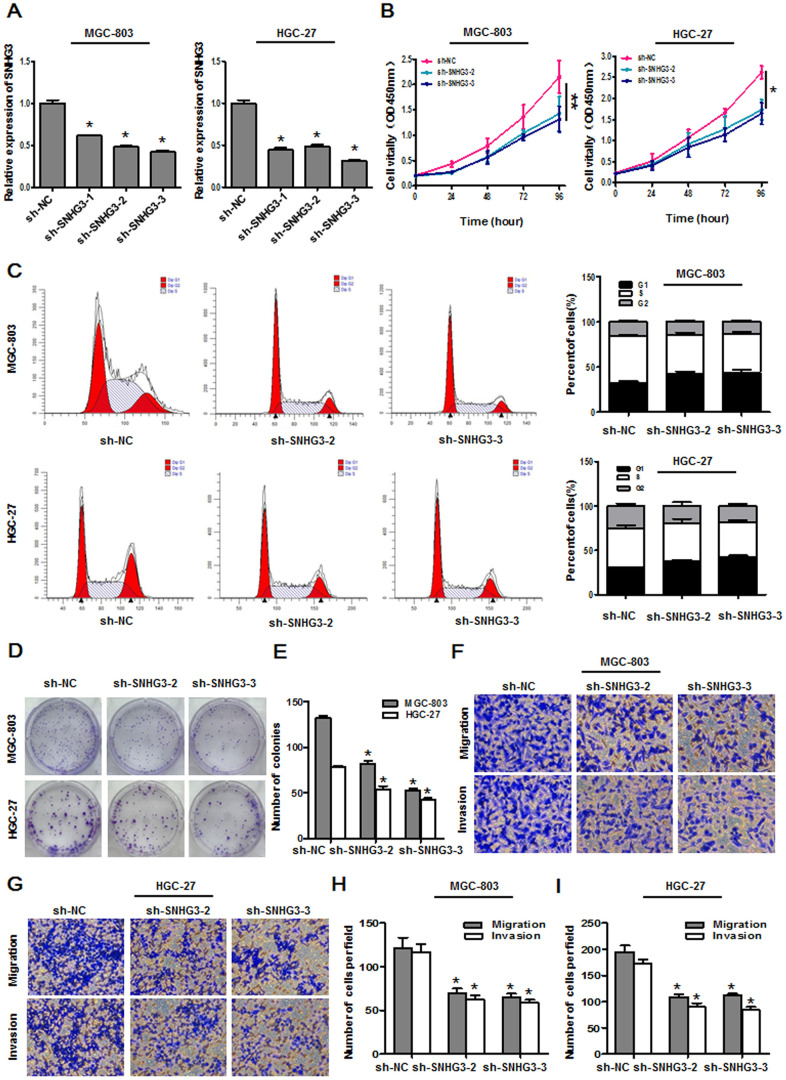
**SNHG3 knockdown suppresses gastric cancer cell growth and metastasis.** (**A**) SNHG3 expression was assessed in MGC-803 and HGC-27 cells following sh-SNHG3 transfection. *P<0.01 vs. sh-NC group. (**B**) Transfected cell proliferation was assessed via CCK-8 assay. *P<0.01, **P<0.05 vs. sh-NC group. (**C**) Cell cycle progression was assessed via flow cytometry following SNHG3 silencing in MGC-803 and HGC-27 cells. (**D, E**) MGC-803 and HGC-27 cell colony formation activity was assessed following SNHG3 silencing. *P<0.01 vs. sh-NC group. (**F**–**I**) Transwell migration and invasion assays were used to assess MGC-803 and HGC-27 cells following SNHG3 silencing. *P<0.01 vs. sh-NC group.

To understand the functional role of SNHG3 in GC cells, we next knocked it down by transfecting MGC-803 and HGC-27 cells with sh-SNHG3 or control constructs, with both sh-SNHG3-2 and sh-SNHG3-3 being able to effectively inhibit the expression of this lncRNA (Figure 2A). A subsequent CCK-8 assay revealed that SNHG3 knockdown suppressed the growth of both of these cell lines relative to sh-NC transfection (Figure 2B). Flow cytometry analyses further confirmed that both of these SNHG3-specific shRNA constructs induced significantly higher rates of cells in the G0/G1 phase and fewer cells in the S and G2 phases relative to control shRNA transfection for both tested cell lines (Figure 2C). Likewise, a colony formation assay indicated that SNHG3 knockdown markedly impaired the clonogenic potential of these GC cells relative to sh-NC transfection (Figure 2D, 2E). These data thus indicated that SNHG3 functions as an oncogene to drive GC cell proliferation.

We additionally assessed the impact of SNHG3 on GC cell migration and invasion via a transwell assay approach, revealing that SNHG3 knockdown significantly impaired the migratory and invasive activities of both cell lines (Figure 2F–2I). This suggests that silencing SNHG3 can compromise the malignant properties of GC cells.

### Overexpressing SNHG3 promotes GC cell growth and metastasis

**Figure 3:**
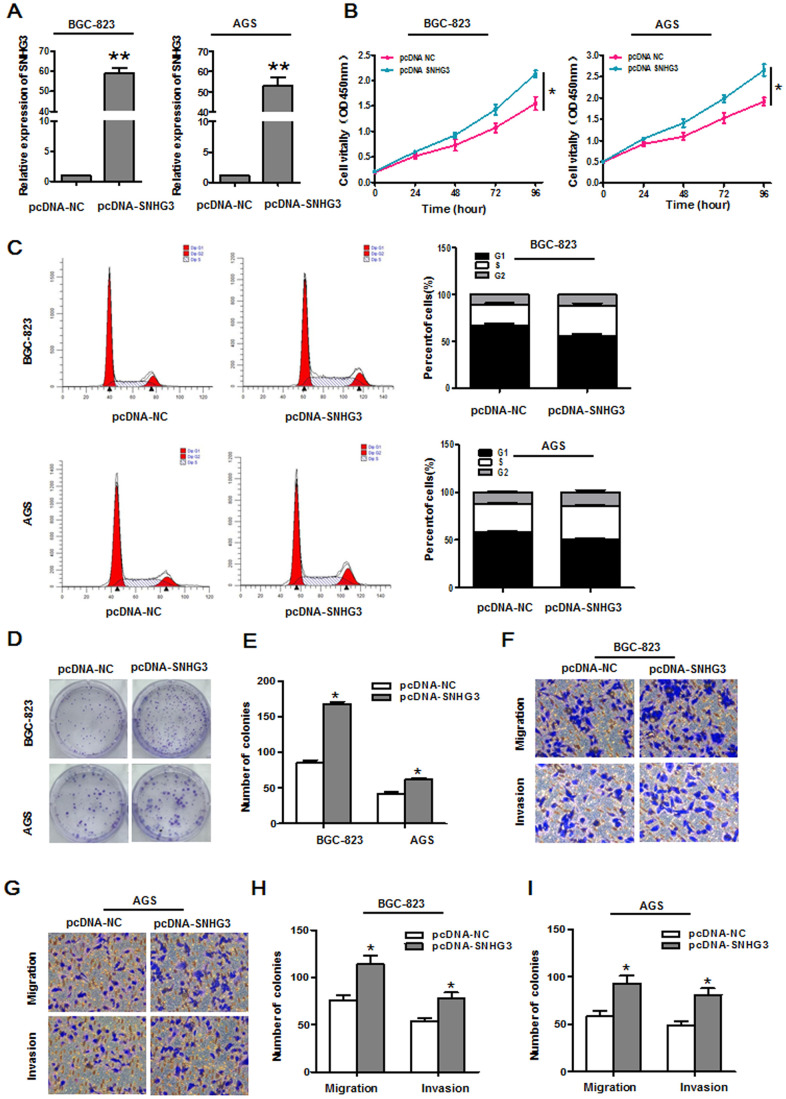
**Overexpressing SNHG3 enhances gastric cancer cell growth and metastasis.** (**A**) SNHG3 expression in BGC-823 and AGS cells was assessed following pcDNA3.1-SNHG3 transfection. *P<0.01 vs. the pcDNA3.1-NC group. (**B**) Transfected cell proliferation was assessed via CCK-8 assay. *P<0.01 vs. the pcDNA3.1-NC group. (**C**) Cell cycle progression in BGC-823 and AGS cells transfected with pcDNA3.1-SNHG3 was assessed via flow cytometry. (**D**, **E**) BGC-823 and AGS cell colony forming activity was assessed following SNHG3 overexpression. *P<0.01 vs. the pcDNA3.1-NC group. (**F**–**I**) Transwell migration and invasion assays were used to assess BGC-823 and AGS cells following pcDNA3.1-SNHG3 transfection. *P<0.01 vs. the pcDNA3.1-NC group.

We next conducted overexpression assays aimed at further confirming the functional role of SNHG3 as a regulator of GC progression by transfecting BGC-823 and AGS cells with the pcDNA3.1-SNHG3 plasmid to upregulate this lncRNA (Figure 3A). Subsequent CCK-8 assays revealed that the overexpression of SNHG3 markedly enhanced BGC-823 and AGS cell proliferation (Figure 3B), while reducing the relative frequency of cells in the G0/G1 phase and increasing the frequency of cells in the S and G2 phases of the cell cycle (Figure 3C). Consistent with these results, SNHG3 overexpression enhanced the colony formation activity for these two GC cell lines (Figure 3D, 3E) and augmented the migratory and invasive activity of these cells in transwell experiments (Figure 3F–3I).

### SNHG3 directly targets miR-139-5p

**Figure 4:**
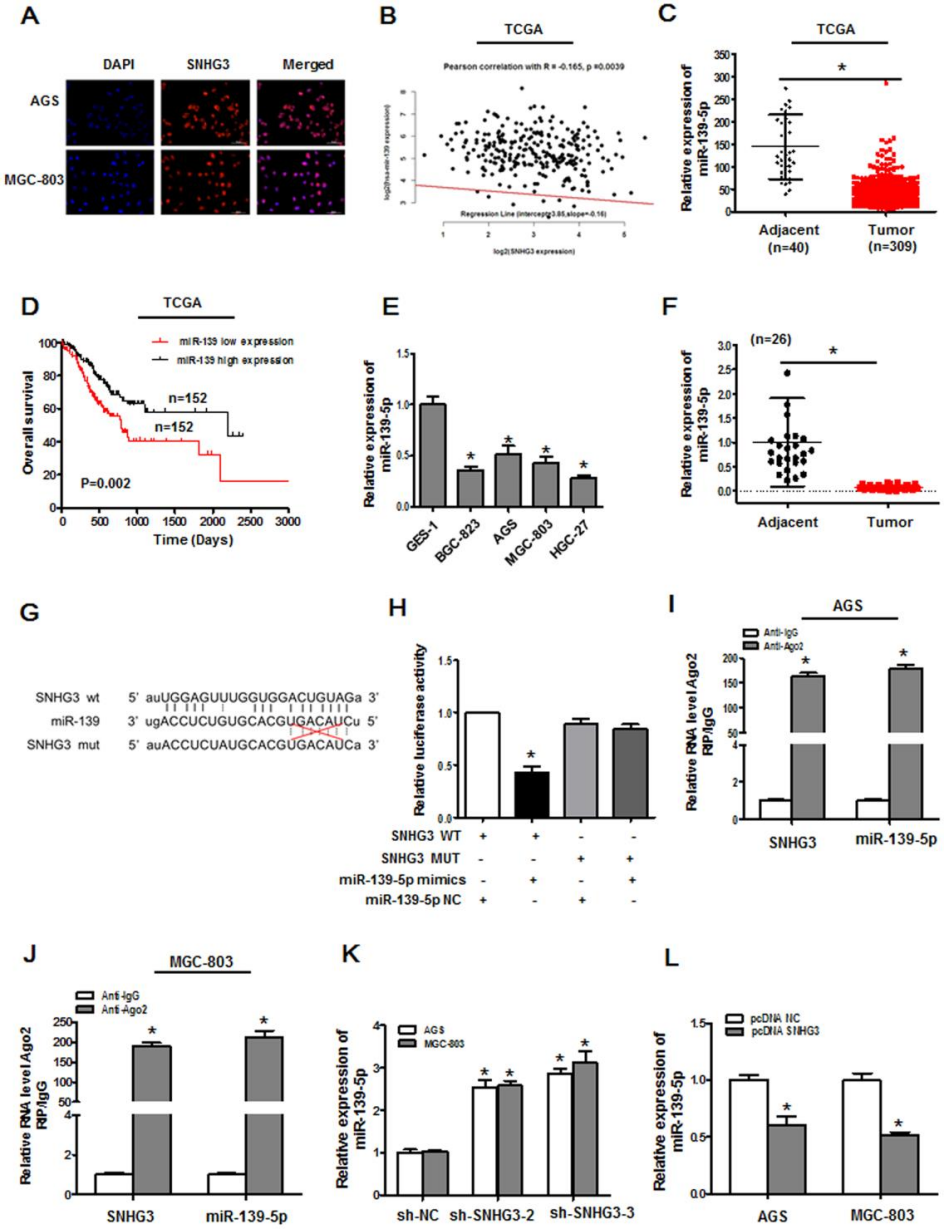
**SNHG3 directly targets miR-139-5p in gastric cancer.** (**A**) The localization of SNHG3 was assessed via an RNA FISH assay in AGS and MGC-803 cells. (**B**) Correlations between the expression of miR-139-5p and SNHG3 in TCGA database samples were assessed via Pearson’s correlation analyses, *R*=-0.165, *P*=0.0039. (**C**) miR-139-5p expression levels in gastric cancer tissues from the TCGA database, *P<0.01. (**D**) The overall survival of gastric cancer patients in the TCGA database was compared as a function of miR-139-5p expression levels (high vs. low; log-rank, *P*=0.002). (**E**) qPCR was used to measure miR-139-5p expression in GES-1, BGC-823, AGS, MGC-803, and HGC-27 cells. *P<0.01 vs. GES-1 cells (**F**) qPCR was used to assess miR-139-5p expression in 26 pairs of gastric cancer tumor and paracancerous tissues, *P<0.01. (**G**) Predicted sequence complementarity between miR-139-5p and SNHG3. (**H**) SNHG3 and miR-139-5p binding in 293T cells was assess via luciferase reporter assay following co-transfection with SNHG3 WT or SNHG3 MUT and miR-NC or miR-139-5p mimics. *P<0.01 vs. control. (**I**, **J**) Interactions between miR-139-5p and SNHG3 were assessed via RIP assays, *P<0.01 vs. the anti-IgG group,. (**K**) miR-139-5p expression was assessed in AGS and MGC-803 cells following sh-SNHG transfection. *P<0.01 vs. the sh-NC group. (**L**) miR-139-5p expression was quantified in AGS and MGC-803 cells following pcDNA3.1-SNHG3 transfection. *P<0.01 vs. the pcDNA3.1-NC group.

To more fully understand how SNHG3 contributes to GC malignancy, we next conducted an RNA-FISH assay to assess its intracellular localization within AGS and MGC-803 cells. This lncRNA was found to primarily localize to the cytoplasm rather than the nucleus (Figure 4A), indicating that it may function by binding to miRNAs and indirectly altering target mRNA expression through a ceRNA mechanism. SNHG3 harbored a predicted miR-139-5p binding region, and that the expression of this miRNA was inversely correlated with that of SNHG3 in the TCGA GC patient dataset (Figure 4B). TCGA analyses further confirmed that marked miR-139-5p downregulation was evident in GC tissues (n = 309) relative to control samples (n = 40) (Figure 4C), with lower levels of miR-139-5p being correlated with GC patient worse overall survival (OS) (Figure 4D). Likewise, we found miR-139-5p to be expressed at lower levels in GC cell lines relative to GES-1 cells and at lower levels in GC tumor tissue samples relative to paired paracancerous tissues as measured via qPCR (Figure 4E, 4F). The putative miR-139-5p binding site within SNHG3 was identified using StarBase (Figure 4G). In luciferase reporter assay, miR-139-5p mimic transfection was sufficient to suppress WT but not mutant SNHG3 luciferase reporter activity (Figure 4H). RIP assays further revealed the significant enrichment of SNHG3 and miR-139-5p in immunoprecipitates generated with anti-Ago2 relative to those prepared using control IgG (Figure 4I, 4J). There were also marked increases or decreases in miR-139-5p expression in AGS and MGC-803 cells following SNHG3 knockdown or overexpression, respectively (Figure 4K, 4L). These data thus revealed that SNHG3 functions as a ceRNA for miR-139-5p in GC cells.

### MYB is a miR-139-5p target gene

**Figure 5:**
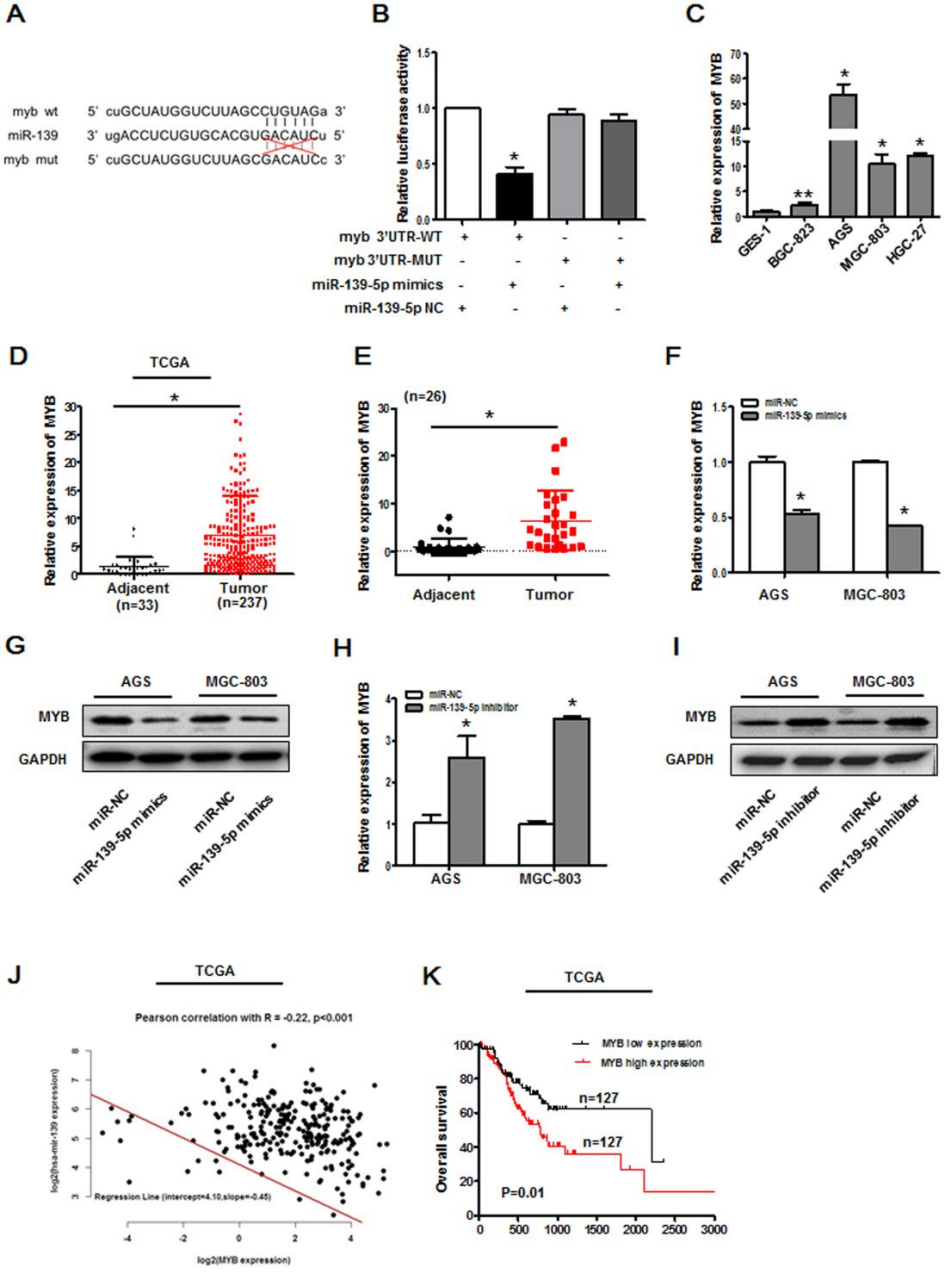
**MYB is a miR-139-5p target gene.** (**A**) Putative MYB and miR-139-5p sequence complementarity as predicted using StarBase. (**B**) Binding interactions between miR-139-5p and MYB were assessed via luciferase reporter assays in 293T cells cotransfected with MYB WT or MYB MUT constructs and miRNA NC or miR-139-5p mimics. *P<0.01 vs. control. (**C**) MYB expression was assessed via qPCR in GES-1, BGC-823, AGS, MGC-803, and HGC-27 cells. *P<0.01, **P<0.05 vs. GES-1 cells. (**D**) MYB expression levels in gastric cancer tissues in the TCGA database, *P<0.01. (**E**) MYB expression levels were assessed via qPCR in 26 pairs of gastric cancer tumors and paracancerous tissues, *P<0.01. (**F**) MYB mRNA levels were assessed in AGS and MGC-803 cells following miR-139-5p mimic transfection. *P<0.01 vs. the miR-NC group. (**G**) MYB protein levels were assessed in AGS and MGC-803 cells following miR-139-5p mimic transfection. (**H**) MYB mRNA levels in AGS and MGC-803 cells were assessed following miR-139-5p inhibitor transfection. *P<0.01 vs. the miR-NC group. (**I**) MYB protein levels were assessed in AGS and MGC-803 cells following miR-139-5p inhibitor transfection. (**J**) Pearson correlation analyses were used to evaluate the relationship between miR-139-5p and MYB expression in the TCGA database, *R*=-0.22, *P*<0.001. (**K**) Overall gastric cancer patient survival in the TCGA database was compared as a function of MYB expression (low vs. high; log-rank, *P*=0.01).

To establish the signaling mechanisms whereby SNHG3/miR-139-5p control GC cell malignancy, we next sought to identify relevant miR-139-5p target genes using the StarBase database and the TCGA GC patient dataset. This analysis revealed a predicted miR-139-5p binding site within the MYB 3’-UTR (Figure 5A). Consistent with this, miR-139-5p mimics were able to significantly suppress MYB-WT luciferase reporter activity in HEK-293T cells (Figure 5B). Notably, GC cells and tissues exhibited pronounced MYB upregulation (Figure 5C–5E). We found that miR-139-5p mimic transfection suppressed MYB mRNA and protein levels in AGS and MGC-803 cells as measured via qPCR and Western blotting, while miR-139-5p inhibitor transfection yielded the opposite phenotype (Figure 5F–5I). The miR-139-5p and MYB expression were negatively correlated *in silico* (Figure 5J), and increased MYB expression was associated with decreased GC patient OS (Figure 5K).

### SNHG3 positively regulates MYB expression by sequestering miR-139-5p

**Figure 6:**
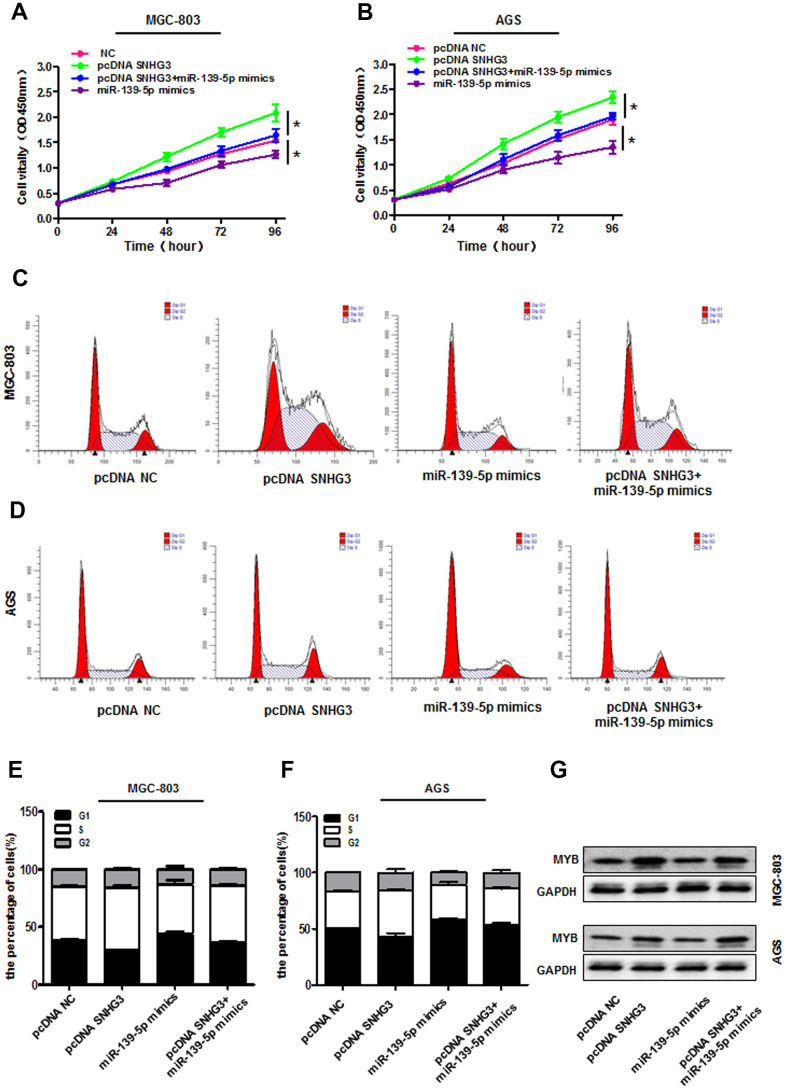
**SNHG3 sequesters miR-139-5p and thereby regulates MYB expression.** (**A**, **B**) AGS and MGC-803 cell proliferation was assessed via CCK-8 assay following pcDNA3.1-SNHG3, miR-139-5p mimic, and pcDNA3.1SNHG3+miR-139-5p mimic transfection. *P<0.01 vs. the pcDNA3.1-NC group. (**C**–**F**) Cell cycle progression in AGS and MGC-803 cells transfected as in (**A**, **B**) was assessed via flow cytometry. (**G**) MYB protein levels in AGS and MGC-803 cells transfected as in (**A**, **B**) were measured.

Given that SNHG3 can function as a ceRNA for miR-139-5p, we next sought to test whether the impact of SNHG3 on GC cell malignancy was attributable to its regulation of this miR-139-5p/MYB axis. In CCK-8 assays, we found that cells overexpressing SNHG3 exhibited significantly improved survival whereas the proliferation of these cells declined slighted upon pcDNA3.1-SNHG3 and miR-139-5p mimic co-transfection in MGC-803 and AGS cells (Figure 6A, 6B). Consistent with this, SNHG3 overexpression drove cell cycle progression from the S stage to the G2 stage, whereas increase arrest at the G0/G1 phase was observed in cells co-transfected with pcDNA3.1- SNHG3 and miR-139-5p mimics (Figure 6C–6F). Western blotting also confirmed that miR-139-5p mimics were able to reverse the SNHG3 overexpression-mediated changes in MYB protein levels in GC cells (Figure 6G). Together, these findings confirmed the ability of SNHG3 to regulate GC cell malignancy via the miR-139-5p/MYB axis.

### LncRNA SNHG3 inhibition suppresses *in vivo* gastric tumor growth

**Figure 7:**
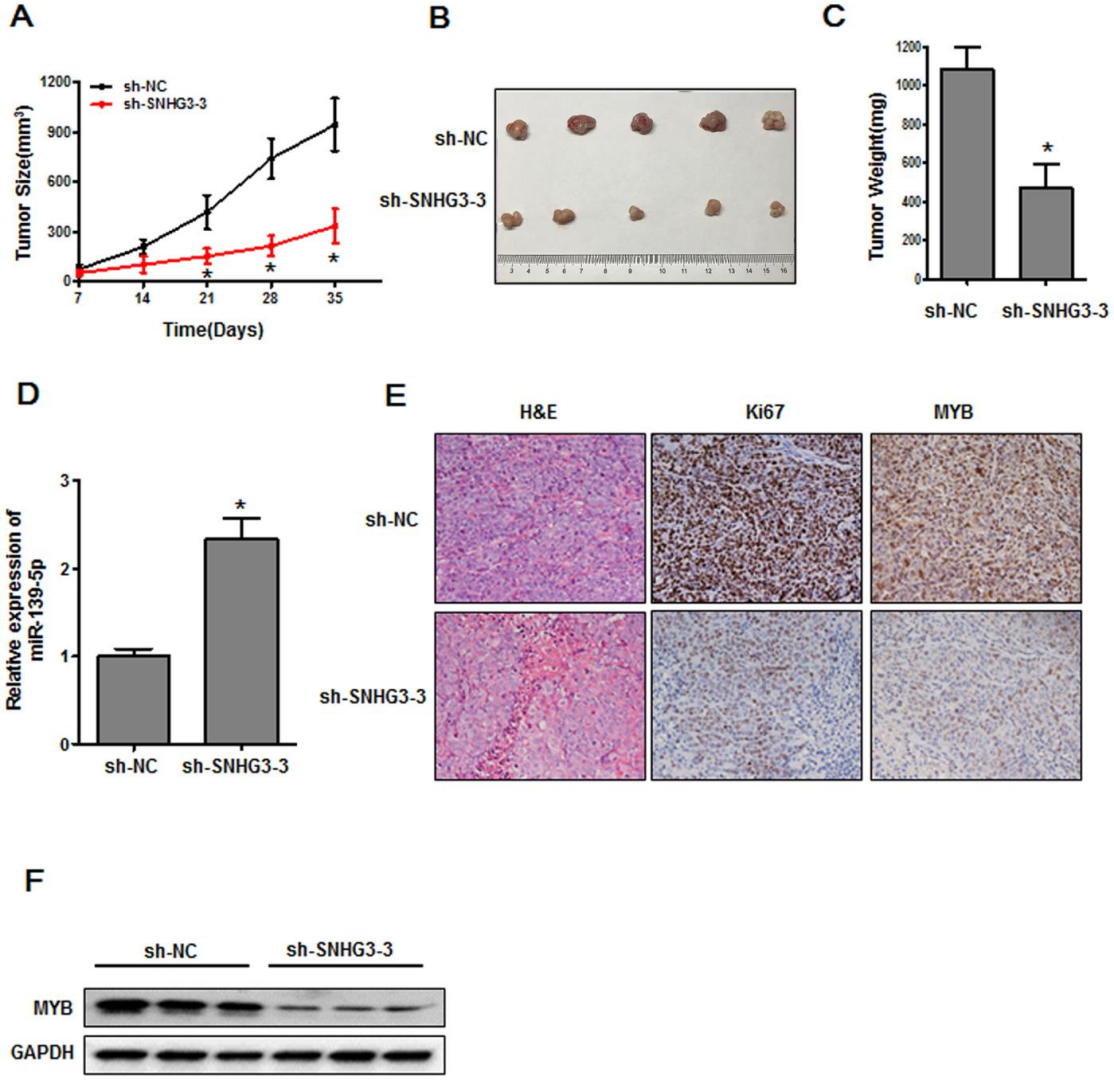
**The inhibition of SNHG3 suppresses *in vivo* tumor growth.** (**A**) Tumor growth in nude mice was monitored, *P<0.01. (**B**, **C**) Tumor size and weight values were measured at the end of the experimental period, *P<0.01. (**D**) miR-139-5p expression was assessed. *P<0.01 vs. the sh-NC group. (**E**) Ki-67 and MYB levels in xenograft tumors were measured via IHC. (**F**) MYB protein levels in xenograft tumors were assessed via Western blotting.

Lastly, we sought to explore the importance of SNHG3 as a regulator of *in vivo* tumorigenesis by implanting nude mice with HGC-27 cells that had been transduced with control or sh-SNHG3 lentiviral vectors in order to establish a xenograft model system. Average tumor weight and volume values were significantly lower in the sh-SNHG3 lentivirus group relative to the control lentivirus group (Figure 7A–7C). The miR-139-5p was expressed at significantly higher levels in tumors harboring the sh-SNHG3 lentivirus relative to control tumors (Figure 7D). IHC analyses further confirmed that SNHG3 knockdown was associated with reduced Ki-67 and MYB protein levels relative to those observed in control tumors (Figure 7E). Western blotting further confirmed that MYB was expressed at significantly lower levels in tumors harboring the sh-SNHG3 lentivirus relative to control tumors (Figure 7F). Together, these data thus confirmed that knocking down SNHG3 was sufficient to impair the *in vivo* growth of GC tumors.

## DISCUSSION

Growing evidence indicates that lncRNAs can influence diverse pathological processes. While lncRNA dysregulation has been closely linked to oncogenesis and cancer progression, the mechanisms governing many of these regulatory relationships remain to be defined. This is particularly true for GC, which is associated with some of the highest worldwide morbidity and mortality rates [3, 22]. To identify lncRNAs that may be involved in the progression of GC, we assessed available TCGA data and identified SNHG3 as being significantly upregulated in GC tumor tissues relative to normal control tissue samples. Such SNHG3 upregulation was also found to be related to GC patient clinical stage and a poorer patient prognosis. Xuan et al. previously demonstrated that SNHG3 can promote GC progression by regulating the methylation status of the MED18 gene [20]. Sun et al. found that the IL-6/STAT3 pathway was able to promote SNHG3 expression whereupon this lncRNA was able to modulate the miR-3619-5p/ARL2 axis to promote stem-like properties in GC cells [21]. SNHG3 is thus a key oncogenic lncRNA in the context of human GC. Even so, the mechanisms whereby it regulates GC metastasis, proliferation, and recurrence remain to be fully defined. Through knockout and overexpression analyses, we found that SNHG3 was able to drive GC cell cycle progression, proliferation, migration, and invasion. We further confirmed these results using a xenograft model system wherein we found that knocking down SNHG3 suppressed tumor growth. Together, our results indicate that SNHG3 thus plays an oncogenic role in the progression of human GC. Given the relationship between this lncRNA and patient clinicopathological characteristics and survival outcomes, it may also represent a potent biomarker that can be used to guide the prognostic evaluation of individuals with GC.

SNHG family lncRNAs are expressed in both the nucleus and the cytoplasm, serving as ceRNAs in tumor cells in the latter compartment [13]. SNHG3 is a key member of this family and a known regulator of the onset and progression of several cancer types [19, 23, 24]. Through FISH assays, we found that SNHG3 was primarily expressed within the cytoplasm of GC cells, suggesting that it may therein function as a ceRNA by sequestering complementary miRNAs and thus driving GC progression. Through bioinformatics analyses, we detected miR-139-5p as a putative SNHG3 target miRNA. Notably, recent work suggests that this miRNA can suppress the growth of bladder cancer [25], GC [26], and colorectal cancer tumors [27]. Consistent with this role, we observed marked miR-139-5p downregulation in GC tissues and cells and found it to be downregulated by SNHG3. Lower miR-139-5p expression was additionally associated with worse GC patient OS, and luciferase reporter and RIP assays confirmed the ability of miR-139-5p and SNHG3 to bind to one another.

The MYB transcription factor can regulate diverse signaling pathways to control the differentiation, survival, and growth of cells such that it is a key mediator of tumorigenesis in many cancer types [28–30]. Liu et al. found that SACC tumor tissues exhibit MYB overexpression which drives the proliferation and metastasis of these tumor cells such that it is a viable target for SACC treatment [31]. Herein, we confirmed that miR-139-5p was able to target and suppress MYB expression in GC cells, with MYB expression ultimately being suppressed by miR-139-5p but upregulated by SNHG3 in this oncogenic setting. Notably, rescue assays indicated that SNHG3 was able to drive malignant activities in GC cells by sequestering miR-139-5p and thereby indirectly promoting MYB upregulation.

In summary, our results suggest that SNHG3 functions in an oncogenic manner to drive GC progression via controlling the miR-139-5p/MYB axis. Given these data and our finding that the expression of this lncRNA was closely associated with GC patient prognosis, this pathway may represent a viable target for the treatment of this deadly disease.

## MATERIALS AND METHODS

### TCGA data analysis

Clinical and RNA expression data pertaining to GC patients were downloaded from the TCGA database (https://tcga-data.nci.nih.gov/tcga/), with lncRNAs being defined as per the coding/non-coding classifications generated by the GENCODE/ENSEMBL pipeline.

### Tissue samples

In total, 26 pairs of GC tumor and paracancerous (>5 cm from the tumor) tissues were obtained from GC patients at the First Affiliated Hospital of Chongqing Medical University between 2018 and 2019. Patients included in this study cohort had not undergone chemotherapy or radiotherapy before sample isolation, and had received a pathological diagnosis of GC. The study was approved by the Research Ethics Committee of Chongqing Medical University and all patient provided informed consent to participate.

### Cell culture

Control human GES-1 gastric mucosa epithelial cells and four GC cell lines (MGC-803, AGS, BGC-823, and HGC-27) were purchased from the Type Culture Collection of the Chinese Academy of Medical Science (Shanghai, China). Cells were cultured in F12K (AGS) or RPMI-1640 (GES-1, BGC-823, MGC-803, and HGC-27) media (Hyclone, MA, USA) containing 10% FBS (Hyclone) and penicillin/streptomycin in a 37° C 5% CO2 humidified incubator.

### qPCR

TRIzol (Invitrogen, CA, USA) was used to extract cellular RNA, after which a PrimeScript RT reagent Kit with gDNA Eraser (Takara, Dalian, China) was utilized to prepare cDNA. All qPCR reactions were performed as per the directions included with TB Green Premix Ex Taq II (Takara, Dalian, China). Relative SNHG3, MYB, and miR-139-5p expression was normalized to that of GAPDH or U6 as appropriate, with primers being as follows: SNHG3 (forward): 5'-GCTTCAGTTCTAAAGGCCCTGAAAC-3' and (reverse): 5'- TCAACCCAGACTTACGGTCCTATTG3'; MYB(forward): 5'-GCCAATTATCTCCCGAATCGA-3' and (reverse): 5'-ACCAACGTTTCGGACCGTA-3'; GAPDH(forward): 5'-GGTCTCCTCTGACTTCAACA-3' and (reverse): GTGAGGGTCTCTCTCTTCCT-3'; miR139-5p: 5'-UCUACAGUGCACGUGUCUCCAGU-3'; U6(forward): 5'-CTCGCTTCGGCAGCACA-3' and (reverse): ACGCTTCACGAATTTGCGT. Analyses were conducted in triplicate, with the 2-ΔΔCt method being used to analyze the resultant data.

### RNA-fluorescence *in situ* hybridization (FISH)

RNA-FISH assays were conducted as per the instructions included in a FISH kit (Ribo, Guangzhou, China). Briefly, AGS and MGC-803 cells were fixed for 15 min using 4% formaldehyde, rinsed with PBS, permeabilized for 5 min with 0.5% Triton X-100 at 4° C, blocked for 30 min with pre- hybridization buffer at 37° C protected from light, air-dried, and incubated overnight with a pre-hybridization buffer solution containing 20 μM of SNHG3 FISH probe (Ribo, Guangzhou, China) at 37° C. After consecutive washes with 4×SSC, 2×SSC, and 1×SSC at 42° C while protected from light, slides were again air-dried, counterstained for 15-min with DAPI, and assessed via fluorescence microscope (Olympus, Tokyo, Japan).

### Transfection

SNHG3 knockdown was achieved using targeted or negative control short hairpin RNA (shRNA) constructs from GenePharma (Shanghai, China). The sh-SNHG3 sequences were: sh-SNHG3-1: 5’-GCAUUUAGCUAGGAAUGCATT-3’; sh-SNHG3-2: 5’- CUAGCAUGAUAGCUUCAGUTT-3’; sh-SNHG3-3: 5’- GGGAUCAUCUAGAAGGUAATT-3’. In addition, a pcDNA3.1 vector encoding SNHG3 and a corresponding empty control vector were purchased from GenePharma (Shanghai, China). All miR-139-5p mimics, miR-139-5p inhibitors, and corresponding controls were from Ribo Bio (Guangzhou, China). Lipofectamine 3000 (Invitrogen) was used as per provided directions to transfect all cells.

### CCK-8 assay

A CCK-8 kit (Dojindo, Tokyo, Japan) was utilized to measure cell proliferation. Cells were added to 96-well plates (2×103/well). After being allowed to adhere, cells were transfected with sh-SNHG3, pcDNA-SNHG3, miR-139-5p mimics, or corresponding controls. Cell growth was then assessed based on provided directions, with absorbance at 450 nm being assessed via microplate reader (Thermo Scientific, MA, USA).

### Colony formation assay

MGC-803 or HGC-27 cells transfected with sh-SNHG3 or sh-NC and BGC-823 and AGS cells transfected with pcDNA3.1-SNHG3 or pcDNA3.1-NC were added to 6-well plates with 500 cells per well in media containing 10% FBS, and were cultured for 14 days with media being replaced every three days. Colonies were then fixed using methanol, stained using 0.25% crystal violet, imaged, and counted.

### Cell migration and invasion assay

To assess migration, cells were serum-starved overnight and added to the upper chamber of a transwell insert without Matrigel (2×10^3^/insert) for 24 h. After this incubation, cells were fixed using 70% methanol, stained with 0.25% crystal violet, and cells on the lower surface in five random fields of view were counted. Cell invasion assay was performed similarly using the insert coated with Matrigel.

### Flow cytometry

Cell cycle progression was assessed by plating cells in 6-well plates (2×105/well). Following transfection, cells were harvested, rinsed with 500 uL of 70% ethanol at -20° C for 24 h, treated for 30 min with RNase A at 37° C, and stained with 5 uL of propodium iodide (KeyGen, Nanjing, China) for 30 min at room temperature protected from light. A Beckman Gallios Flow Cytometer (Beckman Coulter, IN, USA) was then used to analyze these cells.

### Luciferase reporter assay

WT or mutated versions of the full-length SNHG3 sequence and the MYB 3’-UTR were cloned into the pGL3-basic vector (Promega, WI, USA) by GenePharma, yielding the SNHG3-WT, SNHG3-MUT, MYB-WT, and MYB-MUT vectors. HEK-293T cells were transfected with 100 ng of these vectors together with pR-TK (Promega) and miR-139-5p mimic or control vectors (200 nM) via the use of Lipofectamine 3000 (Invitrogen). At 48 h post-transfection, a dual-luciferase reporter assay system (Promega) was utilized to quantify luciferase activity.

### RNA immunoprecipitation (RIP)

RNA co-precipitation was analyzed as per the instructions provided with the Magna RIP RNA-Binding Protein Immunoprecipitation Kit (Millipore, MA, USA) using anti-Ago2 and control IgG antibodies. After precipitation, a qPCR approach was used to measure miRNA and lncRNA expression as above.

### Western blotting

RIPA buffer containing a protease inhibitor cocktail (Beyotime, Jiangsu, China) was used to extract total intracellular protein. Protein samples were then separated via SDS-PAGE, transferred to PVDF membranes (Millipore), and these blots were blocked for 2 h using 5% BSA in TBST. Next, blots were incubated overnight with anti-MYB (1:1000, ab45150, Abcam, MA, USA) or anti-GAPDH (1:1000, bs-0199R, Bioss, Beijing, China) at 4° C. Following incubation with an appropriate secondary antibody, a chemiluminescence detection signal system (Pierce, IL, USA) was employed to detect protein bands.

### Lentiviral transduction

To achieve stable SNHG3 knockdown in GC cells, lentiviral vectors expressing the sh-SNHG3 or corresponding scrambled control sequences were prepared using the pGC-LV-GV287-GFP vector by Sangon Biotech (Shanghai, China) to yield the LV-sh-SNHG3 and LV-NC viruses. These viruses were then used to infect HGC-27 cells at a multiplicity of infection (MOI) of 10 PFU/cell. At 24 h following lentiviral transduction, media was exchanged for fresh virus-free media. After 3 days, GFP expression was used to assess transduction efficiency, and cells were collected for *in vivo* or *in vitro* use.

### Xenograft tumor model experiments

The Animal Experimentation Ethics Committee of Chongqing Medical University approved all animal studies. In total, 10 *BALB/c* nude mice (4-weeks-old) from the Institute of Medical Laboratory Animals, Chinese Academy of Medical Sciences were purchased and housed in specific-pathogen-free conditions in Laboratory Animal Center of Chongqing Medical University with free food and water access for a 7-day period. Mice were then randomized into control and experimental groups (n=5 each). These animals then received a subcutaneous 0.15 mL injection of HGC-27 cells (2×106) infected with LV-NC or LV-sh-SNHG3 in the femoral region. Tumor growth was measured every 7 days using calipers, with tumor growth being calculated as follows: V = 0.5×L×W^2^. After 35 days, mice were then euthanized and tumors were collected, weighed, and utilized for other analyses.

### Immunohistochemistry (IHC)

Collected tumors were fixed using 4% paraformaldehyde (PFA), paraffin-embedded as per primary instructions, and prepared sections were then stained overnight with primary antibodies specific for Ki67 (1:500, ab15580, Abcam, MA, USA) and MYB (1:200, ab45150, Abcam, MA, USA) at 4° C. Sections were then rinsed with PBS, probed for 2 h with an HRP-conjugated secondary antibody, stained with DAB substrate kit (ZSGB-BIO, Beijing, China), and imaged with an appropriate microscope (Olympus).

### Statistical analysis

Data are given as means ± standard deviation (SD), and were compared via Student’s t-tests and one-way ANOVAs. Spearman and Fisher chi-squared tests were used to assess correlations between groups. SPSS 20.0 (SPSS, IL, USA) was employed for all statistical testing, with P<0.05 as the threshold of significance.
